# Supplementary material for: Education from the crib on: The potential of the Newborn Cohort of the German National Educational Panel Study
Source: J Open Psychol Data. 2023 Aug 3;11:13. doi: 10.5334/jopd.81 (PMC12269999; doi:10.5334/jopd.81)
Supplement: Appendix. — Overview of the measured construct in NEPS-SC1. [file jopd-11-81-s1.pdf]

Appendix

Overview of the measured construct in NEPS-SC1

Table A1. Child Characteristics

		Waves
Direct measures[1]	Sensorimotor development	1
	Habituation–dishabituation paradigm (attentional measures)	1,2
	Indicators from parent-child-interaction (behavioural, socio-emotional , cognitive)	1,2,3
	Vocabulary (receptive, PPVT-4[2])	4,6,8
	Working memory (digit span forward or backward)	4,7,8
	Basic cognitive abilities (nonverbal)	
	- Categorization (SON-R Subtest[3])	4
	- Reasoning and perceptual speed (NEPS-test)	7
	Mathematical competence (NEPS test)	5,7,9
	Executive functioning (delay of gratification, flanker task)	4,5,6,8
	Scientific competence (NEPS test)	6,8
	Procedural metacognition (performance judgement)	
	- vocabulary	8
	- mathematical competence	9
	- scientific competence	8
	- reading	9
	Early Reading Comprehension (ELFE II[4])	9
	Reading speed (SLS 2-9[5])	9

Indirect (reported) measures	Sex target child	1
	Date of birth target child	1,2,3,4,5[a]
	Country of birth target child	1
	Citizenship target child	1
	Temperament	
	- subscales IBQ-R[6]	1,2,3
	- subscales CBQ[7]	4*,5*,6*
	- Self-regulation[8]	7*,9
	Child health	1,2,3,4,5,6,7,8,9
	Aspects of child development (cognition, communication, motor, language)	2,3
	Vocabulary (ELFRA[9], parent checklist)	3
	Behaviour	
	- subscales SDQ[10]	4*,5**,6*,7*,8,9
	- aggressive behaviour	8
	Language used at home and institution**	4,5,6,7
	Personality (Big 5)	6,8
	Perceived competencies*	5*,6*,7*,8,9
	Language support**	6,7
	Special educational needs	8
	Coping and motivation	
	- everyday school life	8,9
	- distant learning	9c

Notes. * Information collected from parent and educator **Information collected from educator only

[a] The repeated query of the birth date of the child ensure the correct mapping in the surveys over time.

[1] see Fuß, Gnambs, Lockl & Attig (2021) for more in-depth overviews.

[2] Peabody Picture Vocabulary Test (PPVT-4; Lenhard, Lenhard, Segerer & Suggate, 2015)

[3] Snijders-Oomen Non-verbale Intelligenztest (SON-R 2 1/2 – 7; Tellegen, Laros & Petermann ,2007)

[4] Ein Leseverständnistest für Erst- bis Siebtklässler – Version II (ELFE II; Lenhard, Lenhard & Schneider, 2017)

[5] Salzburger Lese-Screening für die Schulstufen 2-9 (SLS 2-9; Wimmer & Mayringer, 2014)

[6] Infant Behavior Questionnaire - Revised“ (IBQ-R), Very Short Form (IBQ-R-VsF; Helbig, Putnam, Gartstein & Rothbart, 2009; Putnam, Helbig, Gartstein, Rothbart & Leerkes (2014) for validation see: Bayer, Wohlkinger, Freund, Ditton & Weinert, 2015)

[7] Children’s Behavior Questionnaire (CBQ; Rothbart, Ahadi, Hershey & Fisher, 2001)

[8] adapted from the BIKS-study (Elternfragebogen Welle 2; https://www.iqb.hu-berlin.de/fdz/studies/BiKS_3-10)
[9] Elternfragebögen für die Früherkennung von Risikokindern (ELFRA-2; Grimm & Doil, 2006)
[10] The Strengths and Difficulties Questionnaire (SDQ; Goodman, 1997)

Table A2. Learning environment family

		Waves
Socio-demographics/ Structure / distal background aspects	Sex respondent (= interview respondent)	1,2,3,4,5,6,7,8,9
	Sex partner	1,2,3,4,5,6,7,8,9
	Date of birth respondent	1,2,3,4,5,6,7,8,9
	Date of birth partner	1,2,3,4,5,6,7,8,9
	Relation respondent target child	1,2,3,4,5,6,7,8,9
	Siblings	1,2,3,5,6,7,9
	Partnership respondent	1,3,4,5,6,7,8,9
	Relationship partner target child	1,3,4,5,6,7,8,9
	Place of birth/ migration/ citizenship respondent	1,2,3,4,5,6,7,8,9
	Place of birth/ migration/ citizenship partner	1,2,3,4,5,6,7,8,9
	Education respondent	1,2,3,4,5,6,7,8,9
	Education partner	1,2,3,4,5,6,7,8,9
	Employment respondent	1,2,3,4,5,6,7,8,9,9c
	Employment partner	1,2,3,4,5,6,7,8,9
	Household	
	- place of residence	1,2,3,4,5,6,7,8,9
	- members	1,2,3,4,5,6,7,8,9
	- income	1,2,3,4,5,6,7,8,9
	- debts and assets	3
	Parental leave respondent	2,4
	Parental leave partner	2,4
Educationally relevant knowledge, processes/activities, decisions (facets of HLE)	Informedness about	
	- childcare options	1,2
	- educational system	5,7
	Educational decisions	1,2
	Language use in the family and proficiency of family members (German and language of origin)	1,2,3,4,5,6,7,8,9
	Indicators from parent-child-interaction (e.g. sensitivity, stimulation)	1,2,3
	Joint parent-child activities	
	- Various aspects of the home learning environment	1,2,3,4,5,6,7,8,9
	- Focus (domain specific):	
	o Home literacy environment	5,7
	o Reading to child (language used)	3,6,7,8,9
	o Home numeracy environment	6,8
	Co-parenting	2,3

	Parental conflict	2
	Cultural Capital	
	- respondent (activities, number of books)	3
	- respondent (knowledge highbrow culture)	8
	- target child (joint activities)	5
	Parenting practices	
	- Dimensions of parenting style	5,6,7,8,9
	- ICT-related regulations/ parenting	8,9
	Social capital	
	- kindergarten/school	6,9
	- reference group	6,9
	- position generator	7
	Language use media	3,6,9
	Family climate	6
	Language support/speech therapy	6,7,8,9
	Media use target child	6,7,8,9
	Activities outside the home target child	7,8,9
	Domestic activities target child	9
	Lessons in language of origin	8
	Tutoring	9
	Homework and practice	9
	Support with	
	- homework and practice	9
	- distant learning	9c
	Learning opportunities	9c
Orientation – opinions, attitudes, beliefs, aspirations	Perceived cost and benefit of childcare options	1,2
	Educational aspirations (idealistic, realistic)	1,2,4,8
	Migration (cultural orientation)	1,4,6,7,8,9
	Educational goals	2
	Religion	2,7
	Attitudes towards education	4
	Home literacy environment orientation (importance of early skill acquisition)	5,7
	Home numeracy environment orientation (importance of early skill acquisition)	6
	Reasons (respondent) for outside home activities	7,8
	Social trust respondent	7,9
	Political interest respondent	7
	Impression of child’s classroom	

	characteristics respondent	8
	Satisfaction with school respondent	
	Beliefs digital media use respondent	8
	Expectations and worries for future	8,9
	respondent	9c
Health and personality characteristics (respondent)	Health behaviour respondent	1,2
	Health respondent	
	- physical	1,7,8,9,9c
	- mental	1,2,3,7,9,9c
	Personality respondent	
	- Big 5 respondent	5
	- Willingness to take risks respondent	7,9,9c
	- Patience respondent	7
	- Locus of control	9c
	Life satisfaction respondent	7,8,9

Notes. 9c = Information collected in special COVID-19 interview; HLE = Home Learning Environment; ICT = Information and Communication Technologies.

Table A3. Learning environment institution (ECEC/ kindergarten/ school)

Structure	Childcare history (provider, time)*	1,2,3,4,5,6,7,8,9,9c
	Size	2,3,4,5,6,7
	Composition (ethnical, social, disability, sex)	2,3,4,5,6,7
	Staffing	2,3,4,5
	Premises	2,3,4,5
	Sponsors	2,3
	Fees	2,3
	Free places/ waiting list for attendance	2,3,4,5,6,7
	Opening hours	2,3,5
	Socio-demographics educator	2,3,4,5,6
	Admission criteria	2,3,4,5,6,7
	Materials for playing, learning	4,5,6,7
	Socio-demographics staff	4,5,6,7
	School enrolment target child*	7,8,9
	School history target child*	7,8,9
Process	Language use children	2,3,4,5,6,7
	Language use educator	2,3,4,5
	Acclimatization period	2,3
	Communication with parents	2,3,4,5
	Activities with the children	2,3,4,5,6,7
	Language support offer	2,3,4,5,6,7
	Work conditions, experience, further training, supervision educator	2,3,4,5
Orientation	Educational approach	4,5,6,7
	Support focus	4,5,6,7
	Educational plans	4,5,6,7
Additional Characteristics	Health in ECEC	
	- nutrition	2,3,4,5
	- sleep behaviour	2,3
	Kindergarten competition (number kindergartens in the region)	4,5,6,7
	Satisfaction with structural conditions (head of institution)	4,5,6,7

Notes. * Information collected from parent; 9c = Information collected in special COVID-19 interview.

References

Bayer, M., Wohlkinger, F., Freund, J.-D., Ditton, H. & Weinert, S. (2015). *Temperament bei Kleinkindern - Theoretischer Hintergrund, Operationalisierung im Nationalen Bildungspanel (NEPS) und empirische Befunde aus dem Forschungsprojekt ViVA* (NEPS Working Paper No. 59). Bamberg, Germany: Leibniz-Institut für Bildungsverläufe, Nationales Bildungspanel.

Fuß, D., Gnambs, T., Lockl, K., & Attig, M. (2021). *Competence Data in NEPS: Overview of Measures and Variable Naming Conventions (Starting Cohorts 1 to 6). Revised Version 2021.* Bamberg, Germany: Leibniz Institute for Educational Trajectories (LIfBi), National Educational Panel Study.

Goodman, R. (1997). The Strengths and Difficulties Questionnaire: A research note. *Journal of Child Psychology and Psychiatry, 38*(5), 581-586. doi: https://doi.org/10.1111/j.1469-7610.1997.tb01545.x

Grimm, H., & Doil, H. (2006). *ELFRA-Elternfragebögen für die Früherkennung von Risikokindern* (2. überarbeitete Auflage). Göttingen, Germany: Hogrefe.

Helbig, A. L., Putnam, S. P., Gartstein, M. A., & Rothbart, M. K. (2009). *Development and Assessment of Short and Very Short Forms of the Infant Behavior Questionnaire–Revised Society for Research in Child Development. Presentation at the biannual convention of the Society for Research in Child Development*. Denver: CO.

Lenhard, W., Lenhard, A., & W. Schneider (2017). *ELFE II – Ein Leseverständnistest für Erst- bis Siebtklässler*. [ELFE II – A Reading Comprehension Test for First to Seventh Graders]. Göttingen: Hogrefe.

Lenhard, A., Lenhard, W., Segerer, R., & Suggate, S. P. (2015). *Peabody Picture Vocabulary Test (PPVT-4).* German Edition. London: Pearson.

Rothbart, M. K., Ahadi, S. A., Hershey, K. L., & Fisher, P. (2001). Investigations of temperament at three to seven years: The Children's Behavior Questionnaire. *Child development, 72*(5), 1394-1408. doi: https://doi.org/10.1111/1467-8624.00355

Putnam, S. P., Helbig, A. L., Gartstein, M. A., Rothbart, M. K., & Leerkes, E. (2014). Development and assessment of short and very short forms of the Infant Behavior Questionnaire – Revised. *Journal of personality assessment*, 96(4), 445-458. doi: https://doi.org/10.1080/00223891.2013.841171

Tellegen, P.J., Laros, J.A. & Petermann, F. (2007). *Non-verbaler Intelligenztest* (SON-R 2½-7). Göttingen: Hogrefe.

Wimmer, H., & Mayringer, H. (2014). *Salzburger Lese-Screening für die Schulstufen 2–9 (SLS 2–9)*. Bern, Switzerland: Hans Huber.
